# Modified Mitchell-Banks technique in pediatric inguinal hernia repair: a personalized surgical approach

**DOI:** 10.3389/fped.2026.1889280

**Published:** 2026-08-10

**Authors:** Sevgi Büyükbeşe Sarsu

**Affiliations:** University of Health Sciences Türkiye, Gülhane Faculty of Medicine, Gaziantep City Hospital, Department of Pediatric Surgery, Gaziantep, Türkiye

**Keywords:** child, hernia, inguinal, herniorrhaphy, inguinal canal, ultrasonography, postoperative complications, modified mitchell-banks technique

## Abstract

**Background:**

The classical Mitchell–Banks herniotomy (MBH) is widely employed in infants; however, its applicability in older pediatric populations remains incompletely defined. To address this limitation, we describe an external-oblique-sparing modification of MBH incorporating controlled aponeurotic stretching and the Axis-Directed Inguinal Alignment Maneuver (ADIAM), referred to in this study as the Büyükbeşe technique.

**Objective:**

To evaluate the feasibility, short-term clinical outcomes, and early complication profile of the Büyükbeşe technique across different pediatric age groups and to descriptively examine relationships between internal inguinal ring (IIR) diameter, anatomical characteristics, and postoperative outcomes.

**Methods:**

This single-center retrospective study included 110 pediatric patients aged 0–18 years who underwent hernia repair between October 1, 2024, and April 21, 2025. Patients were analyzed according to predefined age groups (0–2, 2–5, 5–10, and 10–18 years), ethnicity, and local surgical complication status.

**Results:**

The mean age was 4.79 ± 4.02 years, and 82.7% of patients were male. The mean IIR diameter was 8.59 ± 4.86 mm. Overall perioperative adverse events occurred in 20.0% of patients, including local surgical complications in 19.1%, predominantly scrotal edema (11.8%) and hematoma (4.5%). One systemic perioperative adverse event, postoperative respiratory failure, occurred in 0.9% of patients and was included in the overall perioperative adverse event count. Patients with local surgical complications were significantly younger (1.31 ± 1.05 vs. 5.61 ± 4.02 years), shorter (71.2 ± 14.2 vs. 98.6 ± 29.8 cm), and had larger IIR diameters than those without local surgical complications (10.88 ± 7.03 vs. 8.05 ± 4.06 mm; *p* = 0.034). Local surgical complication rates decreased with age (0–2 years: 45.7%; 10–18 years: 0.0%; *p* < 0.001). No early recurrence, surgical site infection, or clinically evident testicular complications were observed during the 30-day follow-up period, and all procedures were mesh-free.

**Conclusion:**

In this preliminary technical case series, the Büyükbeşe technique, an external-oblique-sparing modification of Mitchell–Banks herniotomy, was technically feasible in clinical practice, with short-term findings that should be interpreted cautiously because of the observed early local postoperative morbidity. Exploratory observations suggested that younger age, smaller body size, and relatively larger IIR diameters were more frequently observed among patients with local surgical complications; however, these findings remain hypothesis-generating and require prospective validation. The technique may offer preservation of the anterior inguinal wall, but this potential advantage should be considered alongside the technical challenges associated with limited operative exposure, increased traction, and deep tissue manipulation within a confined operative corridor, particularly in the youngest age group. Further evaluation in diverse clinical settings may help clarify the reproducibility and generalizability of this technique.

## Introduction

Inguinal hernia is one of the most common pediatric surgical conditions, with an estimated incidence of 8–50 per 1,000 live births in term infants and up to 20% in extremely low birth weight infants (1). These hernias arise from persistence of a patent processus vaginalis due to failed obliteration after testicular descent (2). A patent processus vaginalis (PPV) is found in approximately 54% of infants under four months of age; however, only 1.2% of PPV-positive infants required hernia repair within the first year of life, and no additional hernias were identified in those followed beyond one year, suggesting that many PPVs remain clinically silent and may resolve spontaneously without surgical intervention (3). Untreated hernias carry a significant risk of incarceration, particularly in infants, necessitating timely surgical intervention (1). Among children requiring inguinal hernia repair, right-sided hernias predominate, accounting for approximately 60% of cases, likely reflecting the later closure of the right processus vaginalis (1, 4).

Pediatric inguinal hernia is primarily diagnosed clinically through history and physical examination, typically presenting as an intermittent groin bulge that becomes more apparent during crying or straining and reduces spontaneously at rest (1). Ultrasonography serves as a useful adjunct in equivocal cases and, although not routinely required in all centers, can provide high diagnostic accuracy for detecting a patent processus vaginalis and assessing hernia defect characteristics while also enabling visualization of inguinal canal anatomy and hernia contents (5, 6).

The optimal timing of surgical repair is particularly important in preterm neonates. Although early repair has traditionally been favored to reduce the risk of incarceration, it is associated with higher rates of perioperative respiratory complications, whereas delayed repair after NICU discharge may provide a safer clinical profile without significantly increasing incarceration risk. These findings support an individualized, patient-specific surgical approach (7). Surgical technique selection is typically guided by patient age. The classical Mitchell–Banks herniotomy (MBH) is widely employed in infants and younger children, although its applicability in older pediatric patients remains incompletely defined. Studies indicate that the elastic nature of pediatric tissues may permit adequate high ligation even without opening the inguinal canal, suggesting that strict age-based technical boundaries may not always be necessary (8).

This uncertainty stems in part from anatomical maturation during growth: as children age, the deep inguinal ring migrates superolaterally, altering the spatial configuration and orientation of the inguinal canal (9, 10). These dynamic anatomical changes may complicate the use of a single standardized technical approach across the pediatric age spectrum.

The technical feasibility and short-term outcomes of MBH in older pediatric patients with more mature inguinal anatomy remain insufficiently characterized. Moreover, external-oblique-sparing modifications of MBH have not been systematically evaluated across a broad pediatric age range. In response to this gap, we introduced an external-oblique-sparing modification of MBH, hereafter referred to as the Büyükbeşe technique. Compared with classical MBH, which primarily relies on tissue elasticity and manual traction for sac exposure, this modification incorporates two technical refinements. First, controlled stretching of the external oblique aponeurosis using a mosquito clamp transiently widens the superficial inguinal ring without incising the aponeurosis. Second, the Axis-Directed Inguinal Alignment Maneuver (ADIAM) uses gentle caudal-to-cranial oscillatory traction along the inguinal canal axis to improve alignment between the internal and external rings.

This axis-guided approach was designed to facilitate visualization and proximal sac dissection in anatomically challenging situations with limited sac exposure while avoiding formal opening of the inguinal canal. The present study aimed to evaluate the feasibility, short-term outcomes, and early complication profile of the Büyükbeşe technique across different pediatric age groups, and to descriptively examine the relationship between internal inguinal ring diameter, anatomical characteristics, and postoperative outcomes.

## Materials and methods

A total of 110 eligible patients were retrospectively analyzed. The sample size was determined by the total number of eligible patients presenting during the predefined study period rather than by an *a priori* power calculation. Because of the retrospective design, no formal sample size calculation was performed. The available cohort was therefore used for descriptive analysis of short-term postoperative outcomes. Accordingly, all analyses were primarily descriptive and exploratory, and findings should be interpreted with appropriate caution within the limitations of this single-center retrospective study.

### Eligibility criteria and data inclusion

Given this retrospective design, all patient records were reviewed for completeness. Patients with missing essential clinical or surgical data were excluded from the analysis. Patients were eligible if they were aged 0–18 years, had a diagnosis of indirect inguinal hernia requiring surgical repair, had a preoperative ultrasonographic measurement of the internal inguinal ring (IIR) diameter, and had complete anthropometric data (height, weight, and body mass index [BMI]). Bilateral hernias were eligible for inclusion and were managed simultaneously during the same operative session, as detailed in the Surgical Technique section. All analyses were performed at the patient level rather than at the hernia-side level.

Exclusion criteria included age >18 years, direct or femoral hernia, recurrent hernia, strangulated or incarcerated hernia, severe uncontrolled systemic disease or organ failure precluding elective surgery, cystic fibrosis, connective tissue disorders, ascites, increased intra-abdominal pressure, history of peritoneal dialysis, inability to undergo surgery or attend follow-up, incomplete medical records, and urogenital anomalies, including concomitant cryptorchidism.

### Data collection and variable definitions

Data were retrospectively retrieved from hospital records. The collected variables included demographic characteristics (age, sex, ethnicity), anthropometric measurements (height, weight, BMI), preoperative ultrasonographic findings (IIR diameter), hernia characteristics (side and type), surgical technique, and postoperative adverse events, including local surgical complications and systemic perioperative adverse events. IIR diameter was measured in millimeters (mm), age in years, height in centimeters (cm), weight in kilograms (kg), and BMI in kilograms per square meter (kg/m^2^).

Because selective internal ring approximation was not recorded as a predefined operative variable in the institutional database, its exact frequency could not be reliably quantified retrospectively and was therefore not included as a separately analyzable variable.

### Ultrasonographic evaluation

Preoperative ultrasonographic measurement of the internal inguinal ring (IIR) diameter was part of the routine clinical workup for all patients presenting with inguinal hernia at our institution during the study period, and these measurements were retrospectively retrieved from medical records. All preoperative ultrasonographic assessments were performed by a single pediatric radiologist with over five years of experience using high-resolution ultrasound equipment. Measurements of the IIR diameter were obtained with the patient in the supine position. Each measurement was performed twice at the widest transverse plane of the internal ring, and the mean value was documented in the medical records. A standardized scanning protocol and consistent device presets were used to improve reproducibility of measurements. Although no formal intra-observer reliability analysis was conducted, the standardized measurement protocol was intended to reduce observer-related variability.

Although the operating surgeon was necessarily aware of the planned surgical approach, ultrasonographic evaluations were performed independently by the pediatric radiologist before surgery and before postoperative outcomes were known. Preoperative IIR measurements were not used as an independent or sole determinant of technique selection; rather, in routine clinical practice, they served as adjunctive anatomical information that could support preoperative anatomical assessment, operative planning, and descriptive interpretation of postoperative outcomes.

### Postoperative follow-up

Postoperative follow-up data were retrospectively obtained from medical records. Clinical evaluations were performed on postoperative day 7 (±1) and day 30 (±2) as part of routine institutional follow-up practice to monitor recovery and detect early postoperative adverse events. These visits were conducted during scheduled outpatient appointments by the same two operating surgeons who performed the index procedures.

Perioperative and postoperative adverse events were recorded and categorized as either local surgical complications or systemic perioperative adverse events. Local surgical complications included surgical site infection, scrotal edema, hematoma, hernia recurrence, iatrogenic undescended testis, symptomatic hydrocele, and testicular atrophy. Systemic perioperative adverse events included postoperative respiratory failure.

In patients with persistent symptoms or abnormal findings, follow-up was extended up to six weeks based on clinical necessity. In cases of suspected recurrence, additional clinical evaluation and imaging were performed when indicated. Patients with incomplete records were excluded per the eligibility criteria; all 110 included patients had complete postoperative records available through the 30-day follow-up period, and no patients were lost to follow-up.

### Outcome definition

For subgroup analyses, the binary outcome variable was the presence of local surgical complications (yes/no) identified in the medical records during follow-up. Overall perioperative complications were reported separately and included both local surgical complications and systemic perioperative complications.

Scrotal edema was defined clinically as visible or palpable scrotal swelling identified during postoperative examination. In cases of persistent or worsening swelling, ultrasonographic evaluation was performed to exclude hematoma, hydrocele, or other underlying pathology.

Surgical site infection was defined as purulent wound discharge, erythema, or wound dehiscence requiring antibiotics, wound care, drainage, or other intervention within 30 days of surgery; no cases were identified in the present series.

Recurrence was defined in the medical records as the presence of a palpable or visible inguinal bulge, particularly when intermittent and reducible. In infants, bulging during crying episodes was also documented. In cases of clinical suspicion, ultrasonographic confirmation was obtained. Sonographic findings suggestive of recurrence included recurrent patency at the operated inguinal canal or a hernia sac containing fluid or bowel content, interpreted together with clinical findings.

Resolution of complications was defined as the absence of clinical signs and symptoms on physical examination at the follow-up visit, including resolution of visible swelling and return to normal scrotal appearance. In cases of persistent symptoms, ultrasonographic evaluation was performed to confirm resolution.

### Surgical technique

Procedures were performed by a pediatric surgical team consisting of two senior surgeons with approximately 20 years of experience in pediatric hernia repair. Both surgeons were well-versed in the Büyükbeşe technique, a modified Mitchell–Banks approach, and had routinely applied it for several years prior to the study, thereby helping to improve procedural consistency and reduce technical variability.

The Büyükbeşe technique was applied using a standardized core operative sequence in all patients to support procedural consistency. This approach was designed to facilitate exposure in anatomically challenging situations, including infants with restricted operative space and older children or adolescents with less favorable inguinal canal alignment, in whom sac exposure with conventional traction alone might be limited.

Contralateral exploration was not performed routinely; instead, it was reserved for patients with clinical suspicion on physical examination, such as a palpable contralateral bulge or hydrocele, in accordance with a selective approach to contralateral exploration. All operations were conducted under general anesthesia, and preoperative bladder emptying was performed to reduce the risk of iatrogenic injury. Bilateral hernias were identified preoperatively (*n* = 5, 4.5%) and repaired simultaneously through two separate inguinal incisions during the same operative session with the Büyükbeşe technique applied identically to both sides.

The inguinal canal was accessed through a skin incision placed just superior and lateral to the pubic tubercle. Camper's fascia was dissected, and the superficial epigastric vessels were gently retracted. Scarpa's fascia was divided using scissors, and hemostasis was achieved with bipolar electrocautery. Minimal bleeding was encountered, consistent with limited superficial dissection.

Following visualization of the external oblique aponeurosis, the Büyükbeşe technique was applied as an external-oblique-sparing modification of the classical Mitchell–Banks technique (MBH). The approach consisted of two principal components. The first was controlled stretching of the external oblique aponeurosis using a mosquito clamp with intermittent, gentle, graded traction to transiently widen the superficial inguinal ring without incising the aponeurosis. Traction was continued only until sufficient widening for adequate operative exposure was achieved, while avoiding excessive tension or visible aponeurotic tearing. The second component was the Axis-Directed Inguinal Alignment Maneuver (ADIAM), which involved gentle oscillatory caudal-to-cranial traction applied to the hernia sac along the inguinal canal axis, with the patient in the Trendelenburg position, to improve alignment between the internal and external rings.

The maneuver was performed intermittently until sufficient ring alignment and proximal sac exposure were achieved, facilitating controlled manipulation and high ligation while attempting to avoid excessive traction or sac injury. (Supplementary Video S1) illustrates identification and exposure of the hernia sac and controlled stretching of the external oblique aponeurosis, whereas (Supplementary Video S2) illustrates application of the Axis-Directed Inguinal Alignment Maneuver (ADIAM). The cremasteric muscle and internal spermatic fascia were carefully separated to expose the hernia sac. The sac was meticulously dissected from the surrounding cord structures—including the vas deferens and gonadal vessels—using fine-tipped Adson forceps. Neither surgical loupes nor intraoperative Doppler was routinely used; identification and protection of the vas deferens and gonadal vessels were achieved through direct visual inspection and careful blunt dissection using standard anatomical landmarks. The vas deferens was not directly grasped at any stage.

After ADIAM-assisted alignment and establishment of an adequate dissection plane, the proximal sac was grasped transversely with a straight mosquito clamp. Gentle cranial traction was then applied while counter-traction was maintained on the spermatic cord, allowing proximal dissection toward the internal ring.

### Sac ligation and selective ring management

In female patients, the hernia sac was opened prior to ligation to assess its contents, given the potential presence of the ovary or fallopian tube. Any adnexal structure was carefully reduced and protected before ligation. Apart from this step, the external-oblique-sparing principles of the Büyükbeşe technique were applied similarly. Upon completion of the dissection, the hernia sac was twisted twice and ligated at the level of the internal inguinal ring (IIR) using a double knot with an absorbable 3-0 or 4-0 polyglactin (Vicryl) suture, after which the redundant sac was excised.

Following sac ligation, repeated ADIAM-assisted alignment facilitated improved operative exposure and aided identification of the medial and lateral fascial margins of the internal ring. Routine internal ring approximation was not performed; however, in selected anatomically challenging cases with persistent marked residual ring dilation, selective internal ring approximation was performed at the surgeon's discretion based primarily on intraoperative assessment of residual dilation, tissue elasticity, and overall anatomical configuration, while larger preoperative ultrasonographic IIR diameters served only as an adjunctive reference rather than an absolute criterion.

When selective internal ring approximation was deemed necessary, the maneuver was performed only after adequate operative exposure had been achieved through repeated alignment maneuvers and blunt dissection. Before suture placement, direct visualization was used to delineate the adjacent gonadal vessels and, in male patients, the vas deferens; these structures were then gently mobilized away from the planned suture path.

The inferior epigastric vessels were identified directly when visible; otherwise, their expected course was defined using the medial border of the internal ring as an anatomical landmark and excluded from the planned needle path. Deep medial or posterior needle passage was avoided, and a deliberately superficial fascial trajectory was maintained to minimize the risk of vascular injury. In male patients, distal traction on the testis toward the scrotum was then applied to maintain elongation of the spermatic cord, thereby keeping the vas deferens and gonadal vessels under gentle traction and away from the intended suture path, while small Ragnell retractors were used to further improve visualization.

When selective internal ring approximation was performed, interrupted 4-0 polyglactin (Vicryl) sutures were placed through the medial and lateral fascial margins of the internal ring using shallow bites. Visualization was maintained as far as permitted by the confined operative corridor, while blind deep bites toward the adjacent cord structures, posterior wall, or expected course of the inferior epigastric vessels were deliberately avoided. No posterior wall dissection or reinforcement was performed in any patient.

### Local infiltration analgesia

Prior to wound closure, local infiltration analgesia was routinely administered into the subcutaneous and aponeurotic layers using lidocaine hydrochloride and bupivacaine hydrochloride. The total infiltrated volume was 1–2 mL, and the actual dose of each agent was calculated according to patient body weight and established maximum safety limits. Lidocaine was kept below 4.5 mg/kg, with a maximum total dose of 300 mg, and bupivacaine was kept below 2–2.5 mg/kg without epinephrine. In infants younger than 6 months, a more conservative bupivacaine dose was used because of immature hepatic metabolism and the increased risk of local anesthetic systemic toxicity.

### Wound closure

Camper's and Scarpa's fasciae were reapproximated using absorbable sutures, and skin closure was performed with subcuticular sutures. No drains were placed in any patient.

### Statistical analysis

Statistical analyses were performed using IBM SPSS Statistics (IBM Corp., Armonk, NY, USA). Continuous variables were expressed as mean ± standard deviation (SD) or median with interquartile range, as appropriate. Normality of data distribution was assessed using the Shapiro–Wilk test. Continuous variables were compared using Student's t-test or the Mann–Whitney U test according to data distribution. For comparisons involving more than two subgroups, one-way ANOVA or the Kruskal–Wallis test was used, as appropriate. Categorical variables were analyzed using Pearson's chi-square test, the likelihood-ratio chi-square test, Fisher's exact test, or the Fisher–Freeman–Halton exact test for contingency tables larger than 2 × 2, as appropriate.

Patients were categorized into four predefined age groups: 0 to <2 years, 2 to <5 years, 5 to <10 years, and 10–18 years. These groups were selected to reflect developmental periods commonly used in pediatric surgical literature, corresponding broadly to infant/toddler, early childhood, middle childhood, and adolescent periods, respectively.

All analyses were performed at the patient level rather than at the hernia-side level. Subgroup comparisons were performed according to age group, ethnicity, and local surgical complication status. Perioperative safety outcomes were reported separately as overall perioperative adverse events and local surgical complications. The patient who developed postoperative respiratory failure was included in the overall perioperative safety denominator and in the overall perioperative adverse event count, but was not classified as having a local surgical complication.

Spearman's rank correlation analysis was used only to evaluate exploratory correlations between IIR diameter and selected continuous variables, including age, BMI, and height. No correlation analysis between IIR diameter and local surgical complication status was included in the final analysis.

Because selective internal ring approximation was not recorded as a predefined operative variable and could not be reliably quantified retrospectively, multivariable regression analyses were not performed. Similarly, ROC-based threshold, prediction, and risk-stratification analyses were not included in the final analysis. All subgroup analyses were exploratory and descriptive in nature and were intended for hypothesis generation rather than confirmatory inference. A *p*-value of less than 0.05 was considered statistically significant.

### Ethical considerations

The study was approved by the Clinical Research Ethics Committee of Gaziantep City Hospital on June 18, 2025 (decision number: 209/2025). As this was a retrospective study, ethics approval for retrospective data review was obtained after completion of the clinical care period and before data extraction and analysis, in accordance with institutional review board policy.

The study covered the period from October 1, 2024, to April 21, 2025. The requirement for written informed consent was waived by the ethics committee due to the retrospective design. Patient confidentiality was strictly maintained through data anonymization and the assignment of unique identifier codes to each case. The study was conducted in accordance with the principles of the Declaration of Helsinki.

## Results

A total of 110 pediatric patients were included in the study. The mean age was 4.79 ± 4.02 years, and 82.7% were male. Of all patients, 60.9% were of Syrian origin and 39.1% were Turkish. The overall mean height and weight were 93.4 ± 29.5 cm and 18.2 ± 13.0 kg, respectively, with a mean BMI of 17.81 ± 3.07 kg/m^2^. Patients with local surgical complications were significantly younger than those without local surgical complications (1.31 ± 1.05 vs. 5.61 ± 4.02 years, *p* < 0.001). They also had significantly lower height (71.2 ± 14.2 vs. 98.6 ± 29.8 cm, *p* < 0.001) and weight (10.2 ± 3.6 vs. 20.1 ± 13.7 kg, *p* < 0.001). No significant difference in BMI was observed between the groups (17.77 ± 3.13 vs. 17.82 ± 3.07 kg/m^2^, *p* = 0.910) (Table 1).

**Table 1:** Demographic, anthropometric, and clinical characteristics, internal inguinal ring diameter, and perioperative and postoperative outcomes stratified by local surgical complication status.

Variable	All patients (*n* = 110)	Local surgical complications (+) (*n* = 21)	No local surgical complications (*n* = 89)	*p*-value
Demographic Characteristics				
Age (years), mean ± SD	4.79 ± 4.02	1.31 ± 1.05	5.61 ± 4.02	<0.001*
Male sex, *n* (%)	91 (82.7%)	18 (85.7%)	73 (82.0%)	0.682
Female sex, *n* (%)	19 (17.3%)	3 (14.3%)	16 (18.0%)	–
Syrian ethnicity, *n* (%)	67 (60.9%)	15 (71.4%)	52 (58.4%)	0.271
Turkish ethnicity, *n* (%)	43 (39.1%)	6 (28.6%)	37 (41.6%)	–
Anthropometric Measurements				
Height (cm), mean ± SD	93.4 ± 29.5	71.2 ± 14.2	98.6 ± 29.8	<0.001*
Weight (kg), mean ± SD	18.2 ± 13.0	10.2 ± 3.6	20.1 ± 13.7	<0.001*
BMI (kg/m^2^), mean ± SD	17.81 ± 3.07	17.77 ± 3.13	17.82 ± 3.07	0.910
Clinical Characteristics				
Right-sided hernia, *n* (%)	74 (67.3%)	15 (71.4%)	59 (66.3%)	0.911
Left-sided hernia, *n* (%)	31 (28.2%)	5 (23.8%)	26 (29.2%)	–
Bilateral hernia, *n* (%)	5 (4.5%)	1 (4.8%)	4 (4.5%)	–
Internal Inguinal Ring Characteristics				
IIR diameter (mm), mean ± SD	8.59 ± 4.86	10.88 ± 7.03	**8.05** **±** **4.06**	0.034*^a^
Perioperative and Postoperative Outcomes, *n* (%)				
Overall perioperative adverse events	22 (20.0%)	21 (100.0%)	1 (1.1%)	–
Local surgical complications	21 (19.1%)	21 (100.0%)	0 (0.0%)	–
Scrotal edema	13 (11.8%)	–	–	–
Hematoma	5 (4.5%)	–	–	–
Hematoma + scrotal edema	2 (1.8%)	–	–	–
Symptomatic hydrocele	1 (0.9%)	–	–	–
Systemic perioperative adverse event: postoperative respiratory failure	1 (0.9%)	0 (0.0%)	1 (1.1%)	–
Recurrence	0 (0.0%)	–	–	–
Surgical site infection	0 (0.0%)	–	–	–
Iatrogenic undescended testis	0 (0.0%)	–	–	–
Testicular atrophy	0 (0.0%)	–	–	–

Complication subtypes are reported for the total cohort only; hematoma + scrotal edema cases were counted as a single combined local complication and were not additionally included in the individual hematoma or scrotal edema subtype totals. One patient developed postoperative respiratory failure and was classified as having a systemic perioperative adverse event. This patient was included in the main safety denominator and in the overall perioperative adverse event count, but was not classified as having a local surgical complication. Therefore, this patient was included in the group without local surgical complications for subgroup comparisons. No cases of early recurrence, surgical site infection, iatrogenic undescended testis, or testicular atrophy were observed during the short-term follow-up period.

SD, standard deviation; BMI, body mass index; IIR, internal inguinal ring.

a IIR diameter was compared using the Mann–Whitney U test.

*Statistically significant at *p* < 0.05.

Bold values indicate statistically significant results (*p* < 0.05).

No patient demonstrated clinical evidence of early recurrence during the 30-day follow-up period; ultrasonographic evaluation was performed when clinically indicated.

Regarding hernia laterality, 67.3% of patients had right-sided hernia, 28.2% had left-sided hernia, and 4.5% had bilateral hernia; no significant association was identified between hernia laterality and local surgical complication status (***p*** **=** **0.911**). All patients underwent hernia repair without mesh.

Overall perioperative adverse events occurred in 22 patients (20.0%). Local surgical complications occurred in 21 patients (19.1%), with scrotal edema being the most frequent local complication (*n* = 13, 11.8%), followed by hematoma (*n* = 5, 4.5%), combined scrotal edema and hematoma (*n* = 2, 1.8%), and symptomatic hydrocele (*n* = 1, 0.9%). All local surgical complications were classified as Clavien–Dindo Grade I and resolved spontaneously during short-term follow-up, typically within two weeks, without complication-specific radiological or surgical intervention. One patient developed postoperative respiratory failure in the early postoperative period; this event was included in the overall perioperative adverse event count as a systemic perioperative adverse event, but was not classified as a local surgical complication.

The overall mean IIR diameter was 8.59 ± 4.86 mm. Patients with local surgical complications had significantly larger IIR diameters than those without local surgical complications (10.88 ± 7.03 vs. 8.05 ± 4.06 mm, *p* = 0.034) (Table 1).

Mean IIR diameter by age group was 9.92 ± 6.18 mm in the 0–2-year group, 8.35 ± 4.22 mm in the 2–5-year group, 7.85 ± 3.95 mm in the 5–10-year group, and 7.50 ± 3.85 mm in the 10–18-year group (*p* = 0.031). Local surgical complication rates by age group were 45.7% in the 0–2-year group, 12.9% in the 2–5-year group, 4.0% in the 5–10-year group, and 0.0% in the 10–18-year group (*p* < 0.001) (Table 2). Within the 0–2-year age group, all patients younger than 1 year (*n* = 6) developed at least one local surgical complication (Table 2).

**Table 2:** Distribution of internal inguinal ring diameter and local surgical complications by age group.

Age Group	*n* (%)	IIR diameter (mm), mean ± SD	Local surgical complications, *n* (%)
0–2 years^a^	35 (31.8%)	9.92 ± 6.18	16 (45.7%)
2–5 years	31 (28.2%)	8.35 ± 4.22	4 (12.9%)
5–10 years	25 (22.7%)	7.85 ± 3.95	1 (4.0%)
10–18 years	19 (17.3%)	7.50 ± 3.85	0 (0.0%)
Total	110 (100%)	8.59 ± 4.86	21 (19.1%)
*p*-value	–	0.031*	<0.001*

The patient with postoperative respiratory failure was included in the total cohort denominator and in the relevant age-group denominator, but was not classified as having a local surgical complication.

IIR, internal inguinal ring.

a Within the 0–2-year age group, all six patients younger than 1 year developed at least one local surgical complication.

*Statistically significant at *p* < 0.05. *p*-values were derived from the Kruskal–Wallis test for IIR diameter and the chi-square or Fisher's exact test, as appropriate, for local surgical complication rates.

Syrian patients were significantly younger than Turkish patients (3.82 ± 3.56 vs. 6.30 ± 4.26 years, *p* = 0.001) and had significantly larger IIR diameters (9.12 ± 5.08 vs. 7.76 ± 4.42 mm, *p* = 0.048). Although local surgical complication rates were numerically higher among Syrian patients than among Turkish patients (22.4% vs. 14.0%), this difference was not statistically significant (*p* = 0.271) (Table 3).

**Table 3:** Age, internal inguinal ring diameter, and local surgical complications according to ethnicity.

Variable	Syrian (*n* = 67)	Turkish (*n* = 43)	*p*-value
Age (years), mean ± SD	3.82 ± 3.56	6.30 ± 4.26	0.001*
IIR diameter (mm), mean ± SD	9.12 ± 5.08	7.76 ± 4.42	0.048*
Local surgical complications, *n* (%)	15 (22.4%)	6 (14.0%)	0.271

The patient with postoperative respiratory failure was included in the relevant ethnicity-group denominator but was not classified as having a local surgical complication.

SD, standard deviation; IIR, internal inguinal ring.

*Statistically significant at *p* < 0.05.

**Figure 1:**
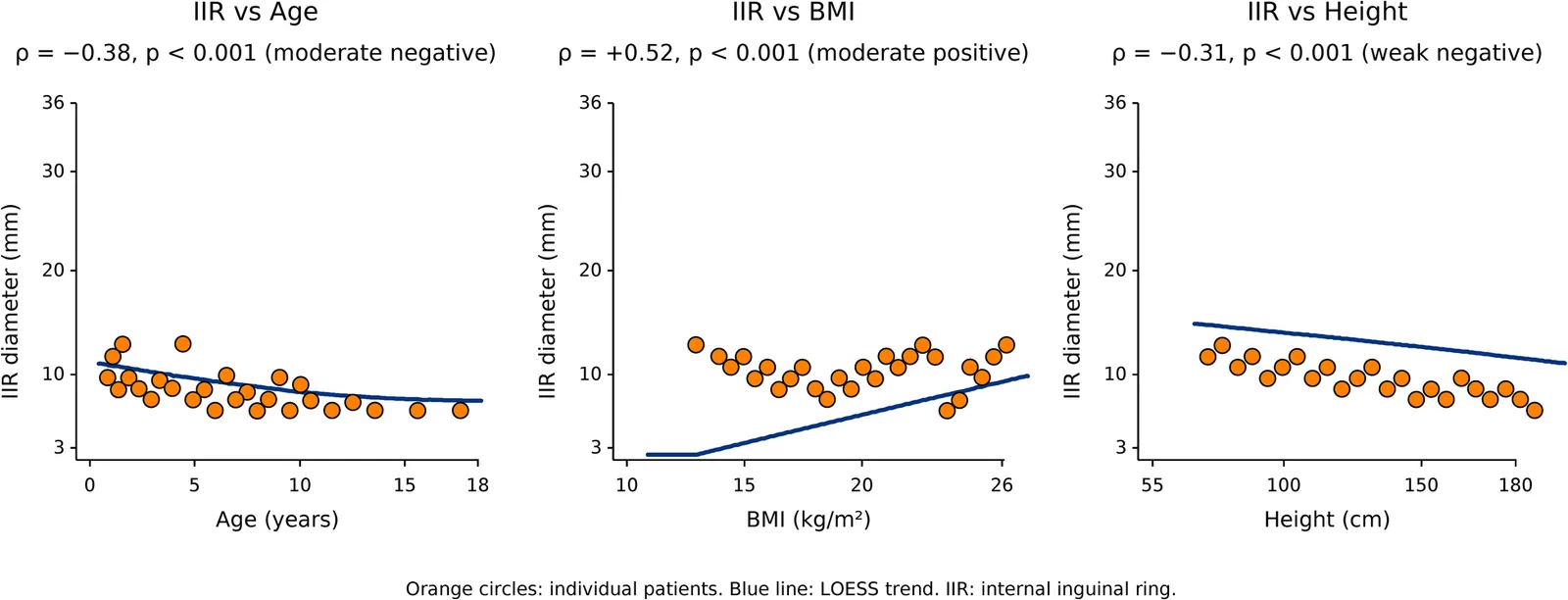
Correlations between internal inguinal ring diameter and continuous age or anthropometric variables. Scatter plots showing the relationship of internal inguinal ring diameter with age, BMI, and height. Spearman's rank correlation analysis demonstrated a negative correlation between IIR diameter and age, a positive correlation between IIR diameter and BMI, and a negative correlation between IIR diameter and height. No correlation analysis between IIR diameter and local surgical complication status is presented.

IIR diameter showed a moderate negative correlation with age (*ρ* = –0.38, *p* < 0.001), a moderate positive correlation with BMI (*ρ* =  + 0.52, *p* < 0.001), and a weak negative correlation with height (*ρ* = –0.31, *p* < 0.001) (Table 4, Figure 1). No correlation analysis between IIR diameter and local surgical complication status is presented.

**Table 4:** Correlation of internal inguinal ring diameter with continuous age and anthropometric variables.

Variable	Spearman correlation coefficient (*ρ*)	*p*-value
IIR vs. Age	−0.38	<0.001*
IIR vs. BMI	+0.52	<0.001*
IIR vs. Height	−0.31	<0.001*

Spearman's rank correlation analysis was used to evaluate correlations between internal inguinal ring diameter and continuous age or anthropometric variables. No correlation coefficient between internal inguinal ring diameter and local surgical complication status is reported in this table. These findings were interpreted as exploratory descriptive associations and were not used to infer independent prediction of local surgical complications.

BMI, body mass index; IIR, internal inguinal ring.

*Statistically significant at *p* < 0.05.

## Discussion

This study evaluates the feasibility and short-term outcomes of the Büyükbeşe technique, an external-oblique-sparing modification of the classical Mitchell–Banks technique for pediatric inguinal hernia repair. The technique combines controlled, fiber-aligned aponeurotic stretching with the Axis-Directed Inguinal Alignment Maneuver (ADIAM), which is intended to temporarily align the internal and external inguinal ring axes and improve anatomical orientation within a confined surgical corridor. By enhancing controlled sac mobilization without formal opening of the inguinal canal, this alignment-based approach was designed to facilitate exposure in anatomically challenging cases, including patients with restricted operative space or increased inguinal canal depth.

Classical Mitchell–Banks herniotomy enables high ligation through the superficial inguinal ring without opening the inguinal canal; however, exposure may remain limited in anatomically restricted cases. In contrast, the Ferguson technique provides broader access but requires formal canal opening (8, 10). The Büyükbeşe technique may therefore offer an intermediate technical approach between classical Mitchell–Banks herniotomy and Ferguson repair. To our knowledge, this specific alignment-based modification has not previously been systematically evaluated across different pediatric age groups.

However, preservation of the external oblique aponeurosis introduces an important technical trade-off. Operating through a confined, unincised corridor may reduce visualization during deep tissue manipulation and narrow the safety margin around the vas deferens, gonadal vessels, and inferior epigastric vessels, particularly when selective internal ring approximation is required. This trade-off may be especially relevant in younger children. In our cohort, the youngest age group demonstrated higher local surgical complication rates, which may reflect smaller anatomical working spaces, more delicate tissue planes, smaller cord-related structures, and reduced tissue-handling margins. Although preservation of the anterior inguinal wall may reduce tissue disruption, the greater traction, aponeurotic stretching, and narrower operative safety margins required in younger children may partly explain the 19.1% early local surgical complication rate observed in the overall cohort and the 45.7% rate observed in the youngest age group, with scrotal edema and hematoma predominating. These findings emphasize that avoidance of external oblique incision should not be interpreted as an unequivocal technical advantage. Rather, this approach should be considered a balance between anterior wall preservation and increased deep tissue manipulation within a limited operative corridor. These observations should be interpreted descriptively, as the retrospective design and limited number of complication events preclude definitive risk stratification or causal inference.

Older children and adolescents may present different technical challenges related to increased canal depth and altered ring orientation, although lower local complication rates were observed in these age groups. These observations suggest that the technical difficulty of the procedure may vary according to developmental anatomy. This suggests that the technical difficulty of the procedure may vary according to developmental anatomy. In younger children, limited working space and delicate tissue planes may increase the risk of traction-related swelling or hematoma, whereas in older children, the main challenge may be deeper dissection and altered alignment between the superficial and internal inguinal rings. These observations remain exploratory and require prospective validation.

A technique using a mosquito clamp to expand the superficial inguinal ring has previously been described in the JOMI surgical video archive (11); however, that approach does not incorporate axis-guided alignment of the inguinal canal. Amato et al. described a distinct approach based on mechanical dilation of the internal inguinal ring, disruption of fibrotic structures, and placement of an intracanal lamellar implant through an open anterior approach (12). The Büyükbeşe technique differs from both approaches by applying axis-guided alignment and controlled aponeurotic stretching to facilitate sac exposure in anatomically challenging cases without formal canal opening or deliberate posterior wall dissection.

Because selective internal ring approximation was not recorded as a predefined operative variable, its exact frequency could not be reliably quantified retrospectively, limiting causal interpretation of the relationship between internal ring size, selective ring approximation, and local surgical complications. High ligation of the hernia sac remains the standard treatment for pediatric indirect inguinal hernia and is generally sufficient in most cases (13). In our cohort, patients who developed local surgical complications had significantly larger internal inguinal ring (IIR) diameters than those without local surgical complications (10.88 ± 7.03 mm vs. 8.05 ± 4.06 mm; *p* = 0.034). This finding should be interpreted as an exploratory descriptive association rather than evidence of independent prediction or causality.

Related findings were reported by Diab et al., who observed recurrence in approximately 10% of children with markedly enlarged IIR diameters (≥12 mm) following high ligation (14). Likewise, Anadolulu et al. reported lower recurrence rates with the Zig maneuver, which includes internal ring narrowing, than with high ligation alone in infants with giant inguinoscrotal hernias, including cases with internal ring diameters ≥2 cm (15). Although these studies primarily evaluated recurrence rather than early postoperative morbidity, their findings are consistent with the possibility that larger IIR diameters may represent more complex inguinal anatomy and may require greater tissue manipulation during sac dissection or, when performed, selective ring approximation.

The age-related variation in IIR diameter and local surgical complication patterns may reflect developmental anatomical differences in the inguinal canal. In the present cohort, IIR diameter decreased significantly with age, measuring 9.92 ± 6.18 mm in the 0–2-year group and 7.50 ± 3.85 mm in adolescents aged 10–18 years (*p* = 0.031).

Correlation analyses similarly showed that IIR diameter was inversely associated with age (*ρ* = –0.38, *p* < 0.001), positively associated with BMI (*ρ* =  + 0.52, *p* < 0.001), and inversely associated with height (*ρ* = –0.31, *p* < 0.001). No correlation analysis between IIR diameter and local surgical complication status is presented, thereby avoiding any implication that IIR diameter represents an independently validated predictor of complications. Weaver et al. reported that approximately 13% of children with asymptomatic patent processus vaginalis subsequently developed symptomatic inguinal hernia, supporting the concept that inguinal canal-related anatomy may evolve clinically during childhood (16).

Syrian patients were significantly younger and had larger IIR diameters than Turkish patients (9.12 ± 5.08 mm vs. 7.76 ± 4.42 mm; *p* = 0.048). Although local surgical complication rates were numerically higher among Syrian patients than among Turkish patients (22.4% vs. 14.0%), this difference was not statistically significant (*p* = 0.271). Therefore, the observed differences in IIR diameter should be interpreted cautiously and may largely reflect differences in age distribution and developmental anatomical characteristics rather than ethnicity itself. These findings are descriptive and should not be interpreted as evidence of ethnicity-related anatomical risk.

Beyond demographic and anatomical differences, the local surgical complication profile warrants consideration. In our series, the Büyükbeşe technique was applied across pediatric age groups ranging from infancy to adolescence, with local surgical complications occurring in 21 patients (19.1%). The most common local surgical complication was scrotal edema (11.8%), followed by isolated hematoma (4.5%), combined scrotal edema and hematoma (1.8%), and symptomatic hydrocele (0.9%). This scrotal edema rate was within the range of mild scrotal edema reported by Diab et al. (10%–31%), whereas more severe and prolonged edema was less frequent in their series (14). Overall, the local surgical complication rate in the present cohort was higher than the combined complication rate of 3.7% reported by Zhang et al. following open repair (17), which may reflect differences in patient age distribution, operative technique, case complexity, and the extent of inguinal tissue manipulation. In a comparative analysis, Ahmad et al. reported higher rates of scrotal edema (29.2%) and hematoma (6.2%) in the Mitchell–Banks herniotomy group than in the Ferguson–Gross herniotomy group (5.4% and 3.1%, respectively), which may partly reflect differences in operative exposure and traction requirements between techniques (8).

One systemic perioperative adverse event, postoperative respiratory failure, occurred in a premature patient with systemic comorbidities. This event was retained in the overall perioperative safety denominator and included in the overall perioperative adverse event count, resulting in an overall perioperative adverse event rate of 22/110 (20.0%), while the local surgical complication rate remained 21/110 (19.1%). Although the event was considered more likely related to prematurity and underlying systemic factors than to the surgical maneuver itself, it was reported separately from local surgical complications. Within the limited 30-day follow-up period, no early recurrence, surgical site infection, iatrogenic undescended testis, or clinically evident testicular atrophy was observed. However, these findings should not be interpreted as evidence of long-term safety or durable recurrence prevention. Although the observed local surgical complications resolved without reintervention, they should not be overlooked as postoperative morbidity and warrant cautious interpretation. Similarly, the limited sample size and short follow-up remain insufficient for reliable assessment of rare surgical, delayed, or systemic perioperative complications, including testicular atrophy, iatrogenic cryptorchidism, recurrence, or anesthesia-related adverse events.

At the other end of the pediatric age spectrum, adolescents represent a unique transitional population between children and adults. Previous reports suggest that mesh-free high ligation may remain feasible in selected adolescent patients. Fan et al. reported no recurrence following laparoscopic high ligation in adolescent indirect hernias with an internal ring diameter of ≤2 cm (18), whereas Taylor et al. reported a 6.0% recurrence rate following open high ligation in adolescents aged 12–18 years and suggested that high ligation may avoid mesh-related morbidity (19). A broader literature review by Lobe et al. found no significant difference in recurrence or complication rates between high ligation and mesh repair in adolescents, supporting a selective individualized approach based on defect characteristics (20). Reistrup et al. emphasized the absence of consensus guidelines for adolescent groin hernia management and noted that continued groin growth may make routine synthetic mesh implantation less desirable in selected patients (21). In our limited adolescent subgroup, the Büyükbeşe technique was feasible, and mesh-free high ligation was technically achievable, with no early recurrence observed during short-term follow-up; however, age-specific treatment implications should be interpreted cautiously. Further prospective comparative studies with longer follow-up are needed to better define recurrence risk, long-term outcomes, and the role of mesh-free repair in this population.

Wu et al. reported iatrogenic cryptorchidism in 0.4% of patients undergoing open inguinal hernia repair, whereas no such complication was observed during short-term follow-up in the present series (22). In our cohort, one patient (0.9%) developed symptomatic hydrocele, which resolved spontaneously without additional intervention. However, given the limited sample size and short follow-up, these findings should be interpreted cautiously and cannot reliably exclude rare surgical, delayed, or anesthesia-related adverse events.

Key technical features of the procedure included avoidance of formal inguinal canal opening, controlled axis-directed exposure, and deliberate protection of adjacent cord structures; when selective internal ring approximation was performed, a superficial fascial needle trajectory was used. However, given the retrospective design, absence of a comparison group, unquantified selective internal ring approximation, and limited follow-up, no conclusions can be drawn regarding relative tissue trauma, relative complication risk, or superiority over conventional open or laparoscopic techniques. Rather, the present findings suggest that the external-oblique-sparing repair was technically feasible without formal opening of the inguinal canal, while the observed early local morbidity requires cautious interpretation.

Age-related surgical considerations remain important in pediatric inguinal hernia repair. Hori and Yasukawa emphasized procedure selection according to patient age, while Nam and Jung highlighted the importance of age-specific surgical strategies (23, 24). In the present series, younger age, shorter height, and larger internal inguinal ring diameter were more frequently observed among patients with local surgical complications and may reflect developmental immaturity and greater anatomical complexity. However, these observations should be interpreted as exploratory descriptive associations rather than evidence of independent prediction or causality. Although preoperative ultrasonographic measurement of IIR diameter is not routinely performed in all centers and pediatric inguinal hernia is often diagnosed clinically, IIR assessment, when available, may provide adjunctive information to support preoperative anatomical evaluation and intraoperative planning. Nevertheless, IIR diameter should not be interpreted as a standalone determinant of surgical technique and should instead be considered together with age, anthropometric characteristics, clinical presentation, and intraoperative findings.

Although genetic and connective tissue factors, including elastin-related abnormalities, have been proposed as contributors to inguinal hernia susceptibility (25, 26), the present study did not evaluate genetic determinants, and their potential relationship to internal inguinal ring morphology remains speculative.

Despite the increasing adoption of laparoscopic and robotic approaches in inguinal hernia repair, optimization of fundamental open surgical techniques remains clinically relevant (27). Access to advanced minimally invasive modalities remains variable worldwide, particularly in resource-limited settings. In this context, surgical modifications that do not require additional equipment or procedural cost may remain relevant, although their reproducibility may depend on surgeon familiarity with the alignment principles, controlled traction, and safety-oriented maneuvers underlying the technique.

Taken together, the present findings suggest that the external-oblique-sparing modification of Mitchell–Banks herniotomy was technically feasible across pediatric age groups, but its short-term postoperative outcomes require cautious interpretation in light of the observed early local morbidity. The technique may preserve the anterior inguinal wall, but this potential advantage must be weighed against increased deep tissue manipulation, traction-related tissue stress, and reduced safety margins around critical cord and vascular structures within a confined, unincised corridor. Accordingly, the present results should be interpreted as preliminary and hypothesis-generating rather than confirmatory. Further prospective comparative studies with standardized recording of selective internal ring approximation and longer follow-up are required to better define the long-term safety, recurrence profile, reproducibility, and clinical role of this technique in pediatric inguinal hernia repair.

### Limitations and future directions

This retrospective single-center study has several limitations. First, the absence of a comparison group precludes direct comparison with conventional open or laparoscopic techniques. Second, the 30-day follow-up period limits assessment of long-term outcomes, including recurrence, testicular ascent, testicular atrophy, persistent hydrocele, chronic pain, and metachronous contralateral inguinal hernia. Although this duration allowed detection of early local complications such as scrotal edema and hematoma, the absence of early recurrence during short-term follow-up should not be interpreted as evidence of durable repair.

Postoperative assessments were performed by the operating surgeons, which may have introduced observer bias. Contralateral exploration was not routinely performed; therefore, the risk of metachronous contralateral inguinal hernia could not be adequately evaluated. Postoperative pain in non-verbal young children was not assessed using validated behavioral scales. The high proportion of Syrian patients reflects the regional demographic characteristics of Gaziantep, which hosts a large refugee population, and may limit generalizability. Potential socioeconomic and healthcare-access differences could not be fully evaluated or accounted for.

The limited number of complication events and the presence of unmeasured confounders may have influenced the exploratory subgroup analyses and should be considered when interpreting observed associations. In addition, the absence of predefined documentation for selective internal ring approximation limited precise retrospective quantification of this adjunctive maneuver and constrained interpretation of technique-specific observations. Accordingly, multivariable regression, prediction, and risk-stratification analyses were not included in the final analysis, and no independent predictor claims were made. The single systemic perioperative adverse event—postoperative respiratory failure—was included in the overall perioperative safety assessment; however, rare anesthesia-related or systemic adverse events cannot be reliably evaluated in this small cohort.

Although most observed local surgical complications resolved without reintervention, they nevertheless represent clinically relevant postoperative morbidity and should be interpreted cautiously. Preservation of the anterior inguinal wall may represent a potential technical advantage; however, this approach may also introduce technical challenges related to limited operative exposure, increased deep tissue manipulation, and the greater traction and aponeurotic stretching required to obtain adequate exposure in anatomically confined operative spaces, particularly in the youngest age group.

The institution-specific availability of routine preoperative IIR measurement may limit the generalizability of anatomical observations in centers where ultrasonographic assessment is not routinely performed. Larger prospective comparative studies with longer follow-up are needed to validate these findings, assess the reproducibility of the Büyükbeşe technique, prospectively document the use of selective internal ring approximation, and clarify how patient-specific anatomical and developmental factors may relate to operative complexity and early postoperative outcomes.

## Conclusion

In this single-center retrospective series, the Büyükbeşe technique, incorporating controlled aponeurotic stretching and the Axis-Directed Inguinal Alignment Maneuver (ADIAM), was technically feasible as an external-oblique-sparing open repair. However, the short-term postoperative outcomes require cautious interpretation in light of the observed early local postoperative morbidity, the occurrence of a systemic perioperative adverse event, and the limited follow-up period. Exploratory observations suggested that early local postoperative morbidity was more frequent among younger children, particularly in the youngest age group; however, these findings remain hypothesis-generating. Although preservation of the anterior inguinal wall may represent a potential technical advantage, this benefit should be considered alongside the technical challenges associated with limited operative exposure, increased traction, and deep tissue manipulation within a confined operative corridor. Further prospective comparative studies with longer follow-up are needed to validate these exploratory observations and better define the role, safety, reproducibility, and generalizability of this technique in pediatric inguinal hernia repair.

## Data Availability

The original contributions presented in the study are included in the article/Supplementary Material, further inquiries can be directed to the corresponding author.

## Supplementary material

Supplementary Video S1 Identification and exposure of the hernia sac, followed by controlled stretching of the external oblique aponeurosis using a mosquito clamp with intermittent, gentle, graded traction to transiently widen the superficial inguinal ring without incising the aponeurosis.

Supplementary Video S2 Application of the Axis-Directed Inguinal Alignment Maneuver (ADIAM), involving gentle oscillatory caudal-to-cranial traction applied to the hernia sac along the axis of the inguinal canal, with the patient in the Trendelenburg position, to improve alignment between the internal and external inguinal rings.
